# In vivo biochemical analyses reveal distinct roles of β-importins and eEF1A in tRNA subcellular traffic

**DOI:** 10.1101/gad.258293.115

**Published:** 2015-04-01

**Authors:** Hsiao-Yun Huang, Anita K. Hopper

**Affiliations:** Department of Molecular Genetics, Center for RNA Biology, The Ohio State University, Columbus, Ohio 43210, USA

**Keywords:** Los1, Msn5, Mtr10, Tef1/2, *Saccharomyces cerevisiae*, nuclear export

## Abstract

Bidirectional tRNA movement between the nucleus and the cytoplasm serves multiple biological functions. To gain a biochemical understanding of the mechanisms for tRNA subcellular dynamics, we developed in vivo β-importin complex coimmunoprecipitation (co-IP) assays using budding yeast. Our studies provide the first in vivo biochemical evidence that two β-importin family members, Los1 (exportin-t) and Msn5 (exportin-5), serve overlapping but distinct roles in tRNA nuclear export. Los1 assembles complexes with RanGTP and tRNA. Both intron-containing pre-tRNAs and spliced tRNAs, regardless of whether they are aminoacylated, assemble into Los1–RanGTP complexes, documenting that Los1 participates in both primary nuclear export and re-export of tRNAs to the cytoplasm. In contrast, β-importin Msn5 preferentially assembles with RanGTP and spliced, aminoacylated tRNAs, documenting its role in tRNA nuclear re-export. Tef1/2 (the yeast form of translation elongation factor 1α [eEF1A]) aids the specificity of Msn5 for aminoacylated tRNAs to form a quaternary complex consisting of Msn5, RanGTP, aminoacylated tRNA, and Tef1/2. Assembly and/or stability of this quaternary complex requires Tef1/2, thereby facilitating efficient re-export of aminoacylated tRNAs to the cytoplasm.

## Abstract (precis)

Huang et al. developed in vivo β-importin complex co-IP assays to study the interactions of β-importins with tRNAs. Los1 (exportin-t) interacts with both unspliced and spliced tRNAs. In contrast, Msn5 (exportin-5) primarily interacts with spliced aminoacylated tRNAs. They demonstrate that Tef1/2 assembles with Msn5–tRNA complexes in a RanGTP-dependent manner.

Eukaryotic tRNAs are transcribed in the nucleus but function in translation in the cytoplasm. However, tRNAs move bidirectionally between the nucleus and the cytoplasm (Shaheen and Hopper 2005; Takano et al. 2005; Zaitseva et al. 2006; Shaheen et al. 2007; Barhoom et al. 2011; Miyagawa et al. 2012). Newly transcribed, end-processed tRNAs are exported from the nucleus to the cytoplasm via primary tRNA nuclear export. Cytoplasmic tRNAs are constitutively imported into the nucleus via retrograde tRNA nuclear import (Shaheen and Hopper 2005; Takano et al. 2005; Zaitseva et al. 2006; Shaheen et al. 2007; Murthi et al. 2010; Barhoom et al. 2011; Miyagawa et al. 2012). The yeast β-importin, Mtr10, is implicated in retrograde tRNA nuclear import (Shaheen and Hopper 2005; Murthi et al. 2010). However, whether Mtr10 acts directly or indirectly in retrograde tRNA nuclear import remains unclear. tRNAs imported into the nucleus from the cytoplasm can again be exported back to the cytoplasm via tRNA re-export (Whitney et al. 2007).

Nuclear export of tRNAs proceeds via the Ran pathway. Ran is a small GTPase that regulates nuclear-cytoplasmic transport via its association with β-importins, also known as karyopherins (Strom and Weis 2001; Fried and Kutay 2003; Madrid and Weis 2006). Exportins are a subset of the β-importins that are dedicated to nuclear export of macromolecules. Due to the asymmetric distribution of Ran GTPase-activating protein (RanGAP) in the cytoplasm and Ran guanine nucleotide exchange factor (RanGEF) in the nucleus (Ohtsubo et al. 1989; Hopper et al. 1990; Bischoff and Ponstingl 1991; Bischoff et al. 1994), exportins cooperatively bind the cargo and RanGTP in the nucleus. After translocation to the cytoplasm, hydrolysis of the RanGTP, aided by the cytoplasmic RanGAP, leads to disassembly of the export complex and delivery of the cargo to the cytoplasm.

In vertebrates, the member of the β-importin family that functions in tRNA nuclear export is exportin-t. Homologs of exportin-t have been studied in budding yeast (Los1), fission yeast (Xpo-t), and plants (PAUSED) (Arts et al. 1998a; Kutay et al. 1998; Hunter et al. 2003; Cook et al. 2009). In vitro biochemical studies of the vertebrate exportin-t and crystallography structural studies of the fission yeast Xpo-t homolog show that exportin-t preferentially binds to the appropriately structured backbone of tRNAs with mature 5′ and 3′ ends, although it has no preference for intron-containing or intron-less tRNAs (Arts et al. 1998b; Lund and Dahlberg 1998; Lipowsky et al. 1999; Cook et al. 2009). Thus, the preferential interaction of exportin-t with appropriately structured tRNAs with mature termini serves as a quality control mechanism that aids end-matured and properly structured tRNAs to preferentially access the cytoplasmic translation machinery.

The *Saccharomyces cerevisiae* exportin-t homolog is Los1 (Hopper et al. 1980; Hellmuth et al. 1998; Sarkar and Hopper 1998). In contrast to vertebrates, the budding yeast splicing machinery is located on the cytoplasmic surface of mitochondria (Yoshihisa et al. 2003, 2007). End-processed intron-containing pre-tRNAs are exported to the cytoplasm prior to intron removal. Because deletion of *LOS1* results in the inhibition of primary tRNA nuclear export, end-processed intron-containing pre-tRNAs, which are unable to access the cytoplasmic splicing machinery, accumulate in *los1Δ* cells (Hopper et al. 1980; Sarkar and Hopper 1998).

Despite the defects of *los1Δ* cells in tRNA nuclear export, *los1Δ* cells are viable (Hurt et al. 1987). *Schizosaccharomyces pombe* Xpo-t and *Arabidopsis* PAUSED are also unessential (Hunter et al. 2003; Cherkasova et al. 2011). Moreover, there is no apparent exportin-t homolog in *Drosophila melanogaster* (Lippai et al. 2000). Because tRNAs play essential roles in the cytoplasm, additional tRNA nuclear export pathways must exist, at least in yeast, *Drosophila*, and *Arabidopsis*.

The vertebrate β-importin family member exportin-5 has also been implicated in the nuclear export of tRNA; however, it is thought to play only a minor role in tRNA nuclear export and instead primarily functions in the nuclear export of pre-miRNAs (Bohnsack et al. 2002; Calado et al. 2002; Lund et al. 2004; Shibata et al. 2006; Mingot et al. 2013; for review, see Leisegang et al. 2012). The yeast homolog Msn5 is well known to export particular phosphorylated transcription factors to the cytoplasm (Kaffman et al. 1998; for review, see Hopper 1999). Msn5 is also involved in tRNA nuclear export because tRNAs accumulate in the nucleus in *msn5Δ* cells (Murthi et al. 2010), and *msn5Δ los1Δ* cells have larger nuclear pools of tRNAs than either *msn5Δ* or *los1Δ* cells (Takano et al. 2005). In contrast to *los1Δ* cells, deletion of *MSN5* does not result in the accumulation of end-processed intron-containing pre-tRNAs (Murthi et al. 2010). One possible explanation for the observed nuclear pools of tRNAs in *msn5Δ* cells is that the nuclear tRNAs in *msn5Δ* cells were previously spliced in the cytoplasm and imported into the nucleus via tRNA retrograde import. Therefore, genetic data support a role for Msn5 in tRNA re-export rather than primary tRNA nuclear export for the subset of tRNAs that are encoded by intron-containing genes (Murthi et al. 2010). However, in vitro studies indicate that Msn5 does not have specificity for mature tRNA, as Msn5 is able to bind to short duplex RNAs (Shibata et al. 2006). To resolve the contradiction between genetic and in vitro biochemical studies, we focused on in vivo biochemical analyses.

Here we developed in vivo β-importin complex coimmunoprecipitation (co-IP) assays to study the in vivo interactions of β-importins with tRNAs. Los1 interacts with both unspliced and spliced tRNAs, whereas we found no evidence for direct interaction of Mtr10 with tRNA. In contrast to Los1, Msn5 primarily interacts with spliced aminoacylated tRNAs. We further demonstrated that Tef1/2, previously implicated in tRNA nuclear export (Grosshans et al. 2000; Bohnsack et al. 2002; Calado et al. 2002; Murthi et al. 2010; Mingot et al. 2013), assembles with Msn5–tRNA complexes in a RanGTP-dependent manner. Our in vivo studies show that Tef1/2 is required to assemble and/or stabilize the Msn5, Tef1/2, aa-tRNA (aminoacyl-tRNA), and RanGTP quaternary complexes.

## Results

### In vivo analyses of tRNA transport complexes

Complexes of β-importins with tRNAs and RanGTP have not yet been reported by in vivo co-IP assays, possibly due to their transient nature (Hellmuth et al. 1998; McGuire and Mangroo 2012). Thus, we developed in vivo β-importin complex co-IP assays to investigate in vivo interactions of β-importins with tRNAs.

RanGTP hydrolysis to RanGDP causes dissociation of exportins from their cargos, and therefore exportins should remain associated with cargo when hydrolysis of RanGTP to RanGDP is inhibited. In contrast, RanGTP dissociates importin–cargo complexes, and therefore these complexes should be enriched when Ran is predominantly in the GDP-bound form. Thus, RanGTP- or RanGDP-locked mutant constructs were used to maintain export or import complexes. Because expression of Ran-locked mutant proteins results in dominant lethality (Kornbluth et al. 1994), Ran constructs encoding RanGTP (Gsp1-G21V)-locked or RanGDP (Gsp1-T24N)-locked mutants were engineered into galactose-inducible expression vectors to induce protein levels in a regulated manner. Los1 and Msn5 maintain their normal subcellular distributions upon 1 h of galactose induction of Ran-locked mutants but alter their subcellular distributions upon longer induction (Huang and Hopper 2014). Therefore, RanGTP- or RanGDP-locked mutants were induced for 1 h in yeast cultures before harvesting cells.

We used β-importins, Msn5, Los1, Mtr10, and Kap95, which are regulated by their endogenous promoters and expressed from yeast episomal (YEp) vectors. MORF (His_6_-HA^epitope^-ZZ^protein A^)-tagged Msn5 (Msn5-M), Los1 (Los1-M), and Mtr10 (Mtr10-M) maintained their biological functions, as assessed by using fluorescence in situ hybridization (FISH) analyses (Supplemental Figs. S1A, S2) and/or complementation of defects of *mtr10Δ* cells (Kramer and Hopper 2013). Thus, the tagged versions of Msn5-M, Los1-M, and Mtr10-M are functional. ZZ^protein A^-tagged Kap95 (Kap95-A), which functions in nuclear import of NLS-containing proteins and has no reported function in RNA nuclear–cytoplasmic trafficking (Enenkel et al. 1995; Gilchrist and Rexach 2003), served as a negative control.

**Figure 1.**
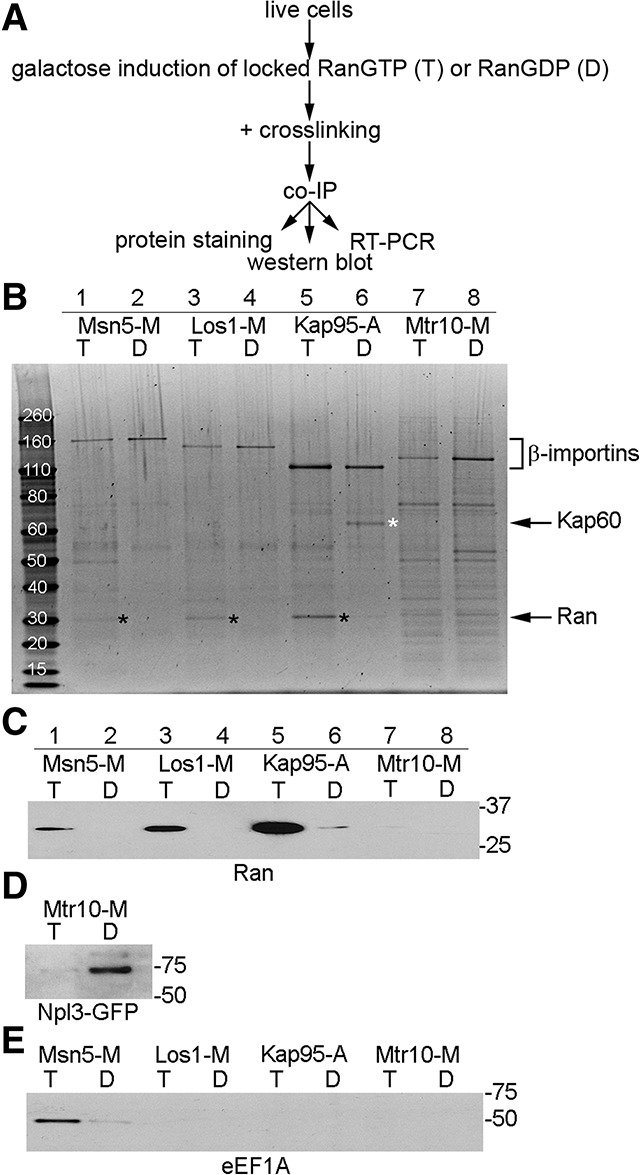
Proteins copurifying with β-importins. (*A*) Flow chart of experimental design. (*B*) Enriched proteins were detected by Sypro Ruby protein staining. (Lanes *1*,*2*) Msn5-M. (Lanes *3*,*4*) Los1-M. (Lanes *5*,*6*) Kap95-A. (Lanes *7*,*8*) Mtr10-M. (Lanes *1*,*3*,*5*,*7*) Cells with RanGTP-locked mutants (T). (Lanes *2*,*4*,*6*,*8*) Cells with RanGDP-locked mutants (D). (Black asterisks) The putative size of Ran; (white asterisk) the putative size of Kap60. (*C*) Enriched Ran was detected by Western blot analyses using anti-Ran antibody. (*D*) To assess binding of Mtr10 with Npl3, strains containing Npl3-GFP and a plasmid encoding functional MORF-tagged Mtr10 (Mtr10-M) were used. Enriched proteins were detected by Western blot analyses using anti-GFP antibody. (*E*) Enriched proteins were detected by Western blot analyses using anti-elongation factor 1α (anti-eEF1A) antibody. (T) Cells with RanGTP-locked mutants; (D) cells with RanGDP-locked mutants.

Formaldehyde cross-linking procedures were used to maintain transient interactions (Fig. 1A). Tagged β-importins were immunoprecipitated using IgG-conjugated magnetic beads. The enriched proteins were analyzed by Sypro Ruby protein staining (Fig. 1B; Supplemental Fig. S3). The proteins ranging from 110 to 165 kDa were the expected size of β-importins (Fig. 1B, indicated by bracket).

Copurified proteins were analyzed by Sypro Ruby protein staining and Western blotting using particular antibodies. A <30-kDa protein copurified with Msn5-M, Los1-M, and Kap95-A (Fig. 1B, black asterisks). This is the expected size of Ran. Indeed, as examined by Western blot analysis, Ran copurified with Msn5-M, Los1-M, and Kap95-A in samples with Ran locked in the GTP-bound state (Fig. 1C, lanes labeled T) but not in samples with Ran locked in the GDP-bound state (Fig. 1C, lanes labeled D). Very little Ran copurified with Mtr10-M under the same conditions, possibly due to its low RanGTP-binding affinity, as previous in vitro studies reported that the affinity of Mtr10 to RanGTP is roughly three orders of magnitude lower than Kap95 (Hahn and Schlenstedt 2011).

Appropriate in vivo interactions for Kap95 were also documented by its association with putative Kap60. Kap95 forms a dimer with Kap60 to mediate nuclear import (Enenkel et al. 1995), and a 60-kDa protein copurified with Kap95-A in a RanGDP-dependent manner (Fig. 1B, white asterisk).

To determine whether tRNAs copurify with the β-importins, enriched RNAs were analyzed by RT–PCR and RT-qPCR. In yeast, 10 tRNA families are transcribed with an intron located 3′ of nucleotide 37. However, many modifications, such as N^1^-methylguanosine, N^3^-methyluridine, or N^3^-methylcytidine, block reverse transcriptase (Wittig and Wittig 1978; Motorin et al. 2007). Since tRNA^Ile^_UAU_ and tRNA^Trp^, encoded by intron-containing genes, and tRNA^Val^_AAC_, tRNA^Gln^_UUG_, and tRNA^His^, encoded by intron-lacking genes, lack nucleotide modifications in the 5′ exon that block reverse transcriptase, they were used to assess the quantity of enriched tRNAs.

### Los1 functions in primary tRNA nuclear export and re-export

**Figure 2.**
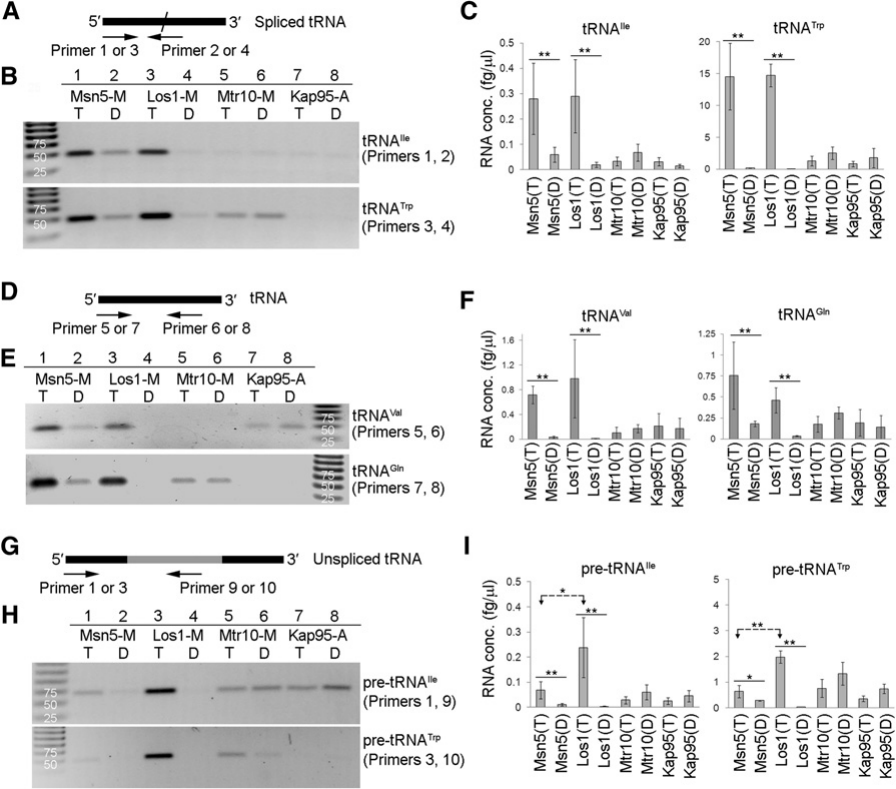
tRNAs copurifying with Los1 and Msn5. (*A*) Spliced tRNAs were detected by reverse transcription using primers crossing the splice junction sequences (primer 2 or 4) and followed by amplification (RT–PCR) using primers 1 or 3 and 2 or 4. (Black lines) Exons; (slash) splice junction. (*B*) RT–PCR analyses for copurified spliced tRNA^Ile^_UAU_ and tRNA^Trp^. (*C*) RT-qPCR analyses for tRNA^Ile^_UAU_ and tRNA^Trp^. (*D*) Primers 5 and 6 or 7 and 8 were used to detect tRNA^Val^ or tRNA^Gln^. (Black line) Exon. (*E*) RT–PCR analyses for enriched tRNA^Val^ and tRNA^Gln^. (*F*) RT-qPCR analyses for tRNA^Val^ and tRNA^Gln^. (*G*) Unspliced tRNAs were detected by reverse transcription using primer 9 or 10 and then amplified by using primers 1 and 9 or 3 and 10. (Black lines) Exons; (gray line) intron. (*H*) RT–PCR analyses for enriched pre-tRNA^Ile^_UAU_ and pre-tRNA^Trp^. (*I*) RT-qPCR analyses for pre-tRNA^Ile^_UAU_ and pre-tRNA^Trp^. Student's *t*-test was used to calculate statistical significance for RT-qPCR reactions. (**) *P* < 0.005; (*) *P* < 0.05. (T) Cells with RanGTP-locked mutants; (D) cells with RanGDP-locked mutants.

As assessed by RT–PCR using primers specific for spliced tRNA^Ile^_UAU_ and tRNA^Trp^ (Fig. 2A, primers 1, 2 or 3, 4), these tRNAs copurified with Los1-M in cells with Ran in the GTP-locked form but not in the GDP-locked form (Fig. 2B, lanes 3,4). Three experiments from independent yeast cultures were used for all RT-qPCR analyses. To obtain the absolute levels of enriched tRNAs, we generated standard curves for the tested tRNAs. RT-qPCR analyses confirmed that Los1 interacts with spliced tRNAs. *P*-value obtained by Student's *t*-test documented the statistical significance (Fig. 2C). We quantified the ratio of tRNA levels in the enriched β-importin complex with locked RanGTP to that with locked RanGDP, resulting in a RanGTP/RanGDP ratio (Table 1, *R*). If the β-importin interacts with tRNA in a RanGTP-dependent manner, one would expect that the *R* ratio for this β-importin would be higher than the *R* ratio for the negative control Kap95-A. Indeed, the *R* ratio for Los1-M, ranging from 14.66 to 281.70, is much higher than the *R* ratio for the Kap95-A sample (*R*_kap95_, 0.47–2.27) for spliced tRNAs (Table 1). Los1-M also interacts with tRNA^Val^ and tRNA^Gln^ encoded by intron-lacking genes in a RanGTP-dependent manner (Fig. 2D–F; Table 1). Thus, Los1 in complex with RanGTP binds mature/spliced tRNAs to form nuclear export complexes.

**Table 1.**
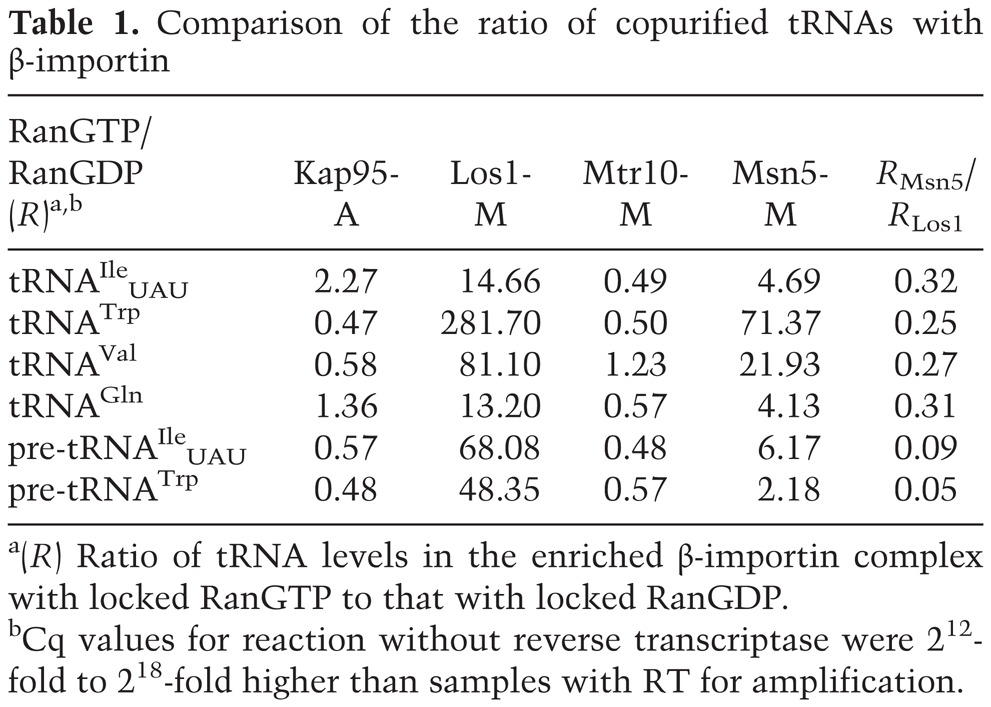
Comparison of the ratio of copurified tRNAs with β-importin

To determine whether Los1 is able to interact with pre-tRNAs that contain introns, the RT–PCR amplifications were conducted with primers that specifically amplify unspliced pre-tRNA^Ile^_UAU_ and pre-tRNA^Trp^ (Fig. 2G, primers 1, 9 or 3, 10). The data show that unspliced pre-tRNA^Ile^_UAU_ and pre-tRNA^Trp^ copurified with Los1-M when cells contained RanGTP-locked mutant proteins but not when cells contained the RanGDP-locked forms (Fig. 2H, lanes 3,4), as supported by using RT-qPCR to quantify unspliced tRNAs (Fig. 2I; Table 1). Therefore, spliced/mature and unspliced tRNAs assemble with Los1 in a RanGTP-dependent manner.

### Mtr10 may function indirectly in tRNA retrograde import

Mtr10 is implicated in retrograde tRNA nuclear import, as *mtr10Δ* cells fail to accumulate nuclear tRNAs upon amino acid deprivation (Shaheen and Hopper 2005; Murthi et al. 2010). Unlike *los1Δ* and *msn5Δ* cells, which accumulate nuclear tRNAs, *mtr10Δ los1Δ* and *mtr10Δ msn5Δ* cells do not accumulate nuclear tRNAs (Murthi et al. 2010). The data provide support for the model that Mtr10 functions upstream of Msn5 in retrograde tRNA nuclear import (Murthi et al. 2010).

We used the co-IP assays to address whether Mtr10 functions directly to import tRNAs into the nucleus. Mtr10 is known to function as the Npl3 nuclear import receptor (Senger et al. 1998). Copurification of Npl3-GFP with Mtr10-M in a RanGDP-dependent manner provides evidence for the interaction of Mtr10 with its known import cargo, as assessed by the co-IP assays (Fig. 1D). In contrast to the predictions, RT–PCR showed that similar low levels of tRNAs copurified with Mtr10-M in extracts with either RanGTP- or RanGDP-locked mutants (Fig. 2B,E,H [lanes 5,6], C,F,I). Similar low levels of tRNAs were also detected in the negative control Kap95-A (Fig. 2B,E,H [lanes 5–8], C,F,I). The *R* ratio for Mtr10-M is similar to the *R* ratio for Kap95-A (Table 1). The amplified signals for RNAs copurifying with Mtr10-M are likely due to contaminating RNAs from the cell extracts. Although Npl3-GFP specifically copurified with Mtr10-M, in the same experiments, no statistically significant amount of tRNAs copurified with Mtr10-M. Thus, the co-IP assays provided no evidence for direct interaction of Mtr10 with tRNAs.

### Msn5 preferentially interacts with mature/aminoacylated tRNAs

Unlike *los1Δ* cells, cells with deletion of *MSN5* do not accumulate intron-containing pre-tRNAs (Murthi et al. 2010). To assess whether Msn5 plays only a minor role in primary tRNA nuclear export, we assessed whether elevated levels of Msn5 could suppress the *los1Δ* phenotype of accumulation of end-processed intron-containing pre-tRNAs. *los1Δ* cells harboring fourfold or 57-fold elevated levels of active, tagged, or untagged Msn5 still accumulated end-processed intron-containing pre-tRNAs (Supplemental Fig. S1B–E), demonstrating that overexpressed Msn5 is unable to suppress the defects of *los1Δ* cells. These genetic data support the model that Msn5 participates in tRNA nuclear re-export of previously cytoplasmic spliced tRNAs but not in primary tRNA nuclear export of intron-containing pre-tRNAs. However, previous in vitro studies reported that Msn5 does not have specificity for mature tRNA, as Msn5 is able to bind to short dsRNAs (Shibata et al. 2006). Thus, we used the co-IP assays to investigate the in vivo role of Msn5 in tRNA nuclear export.

RT–PCR analyses showed that spliced tRNA^Ile^_UAU_ and tRNA^Trp^ as well as mature tRNA^Val^ and tRNA^Gln^ copurified with Msn5-M from extracts with Ran locked in the GTP-bound state, whereas only low levels of tRNAs copurified from extracts with Ran locked in the GDP-bound state (Fig. 2B,E, lanes 1,2). RT-qPCR analyses supported the visual assessment. The levels of enriched spliced tRNAs in Msn5-M and Los1-M samples with locked RanGTP were nearly identical (Fig. 2C). Although low levels of tRNAs were detected in Msn5-M samples with the RanGDP-locked form, these levels were similar to the levels in the negative control Kap95-A (Fig. 2C). In contrast to Los1-M, low levels of unspliced tRNAs copurified with Msn5-M (Fig. 2H,I). Comparison of the levels of enriched tRNA with Msn5-M with that with Los1-M showed that for spliced tRNA^Ile^_UAU_, the *R* ratio for Msn5-M is ∼32% of the ratio for Los1-M (Table 1). In contrast, for pre-tRNA^Ile^_UAU_, the *R* ratio for Msn5-M is ∼9% of the ratio for Los1-M. Similarly, for mature tRNA^Trp^, the *R* ratio for Msn5-M is 25% of the ratio for Los1-M, whereas for pre-tRNA^Trp^, the *R* ratio for Msn5-M is only ∼4% of the ratio for Los1-M. Thus, although Msn5-M appears to interact less well with mature tRNAs than Los1-M, it interacts exceedingly poorly with intron-containing pre-tRNAs. One caveat is that these comparisons are highly influenced by detectable RNAs from extracts with locked RanGDP, as the levels of detectable RNAs in Msn5-M samples with locked RanGDP are higher than the levels in Los1-M samples. Taken together, compared with Los1, Msn5 preferentially binds to tRNAs lacking introns.

**Figure 3.**
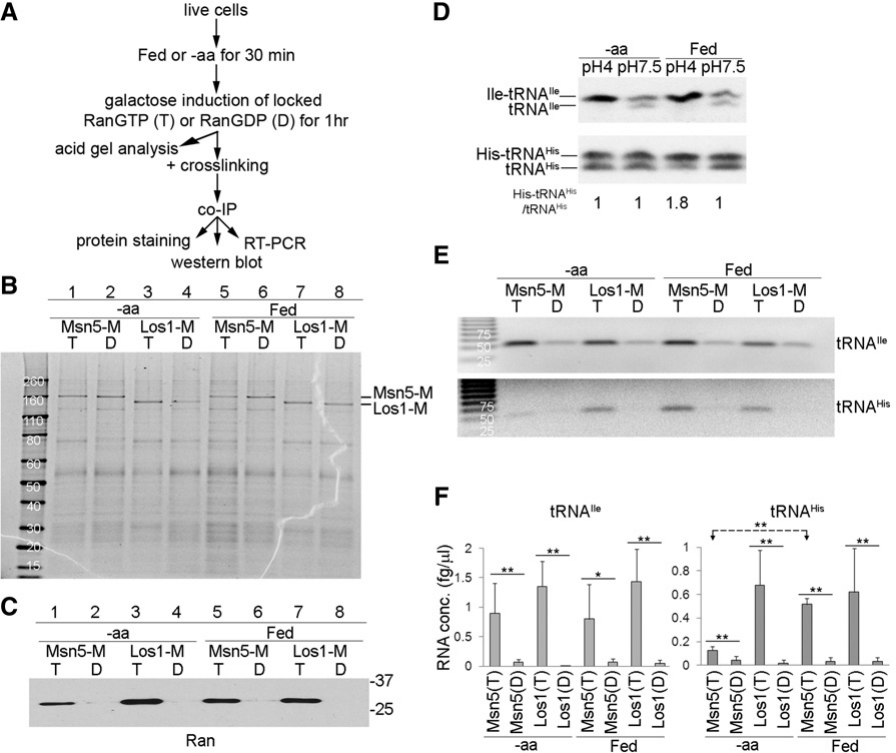
Msn5 preferentially interacts with aminoacylated tRNAs. (*A*) Flow chart of experimental design. (*B*) Enriched proteins were detected by Sypro Ruby protein staining. (Lanes *1*,*2*,*5*,*6*) Msn5-M. (Lanes *3*,*4*,*7*,*8*) Los1-M. (Lanes *1*,*3*,*5*,*7*) Cells with RanGTP-locked mutants (T). (Lanes *2*,*4*,*6*,*8*) Cells with RanGDP-locked mutants (D). (*C*) Enriched proteins were detected by Western blot analyses using anti-Ran. (*D*) Acid gel analyses for aminoacylated and uncharged tRNAs. tRNAs were isolated from cells incubated in medium containing all amino acids or medium lacking all amino acids for 1.5 h. Cells were lysed using either standard pH buffers or pH 4.5 buffers to stabilize the aminoacyl bond. Aminoacylated tRNAs migrate slower than uncharged tRNAs, as indicated at the *left*. (*E*) RT–PCR analyses for tRNA^Ile^_UAU_ and tRNA^His^ copurifying with Msn5-M or Los1-M under starved and fed conditions. (*F*) RT-qPCR analyses for copurified tRNA^Ile^_UAU_ and tRNA^His^. Student's *t*-test was used to calculate statistical significance of the RT-qPCR amplifications. (**) *P* < 0.005; (*) *P* < 0.05. (T) Cells with RanGTP-locked mutants; (D) cells with RanGDP-locked mutants.

Previous studies indicated a role for aminoacylation of tRNAs in the nucleus in tRNA nuclear export (Lund and Dahlberg 1998; Sarkar et al. 1999; Grosshans et al. 2000; Gu et al. 2005). We tested whether the tRNAs that interact with Msn5 are aminoacylated. Yeasts are able to produce all 20 amino acids unless they harbor mutations of particular amino acid biosynthesis pathways. Since the yeast strains that we used are auxotrophic only for histidine, by depleting all amino acids in the media, we could create a situation in which all tRNAs are charged except for tRNA^His^ (Fig. 3A). tRNA^Ile^_UAU_ is aminoacylated when cells are provided amino acids as well as upon amino acid deprivation (Fig. 3D, top). The majority of tRNA^His^ is also aminoacylated when it is isolated from cells provided with all amino acids. However, deprivation of amino acids for 1.5 h resulted in a reduced pool of charged tRNA^His^ for this histidine auxotrophic strain (Fig. 3D, bottom).

If Msn5 has a preference for charged tRNAs, one would expect a reduction of tRNA^His^ copurifying with Msn5 when cells cannot synthesize histidine, and amino acids are unavailable. Msn5 and Los1 complexes were enriched from cells incubated in medium with or without amino acids for 1.5 h (Fig. 3B). Copurification of Ran with Msn5-M and Los1-M documented that export complexes were maintained in both conditions (Fig. 3C). Assessment of RT–PCR showed that tRNA^Ile^_UAU_, which remains charged upon amino acid removal, copurified with Msn5-M under both fed and amino acid-deprived conditions. Although tRNA^His^ copurified with Msn5-M under fed conditions, the levels were considerably reduced when amino acids were removed from the medium (Fig. 3E). As supported by RT-qPCR, the levels of enriched tRNA^Ile^_UAU_ from Msn5-M samples with locked RanGTP under both fed and amino acid-deprived conditions were similar. Compared with fed conditions, the levels of tRNA^His^ that copurified with Msn5-M upon amino acid removal were reduced fourfold (Fig. 3F). The levels of enriched tRNA^Ile^_UAU_ and tRNA^His^ from Los1-M samples with locked RanGTP under both fed and amino acid-starved conditions were similar (Fig. 3E,F). Thus, the data provide evidence that Los1 interacts with both charged and uncharged tRNAs, whereas Msn5 preferentially interacts with aminoacylated tRNAs.

### Msn5 interacts with Tef1/2 in vivo

The in vivo co-IP studies provide evidence that Msn5 preferentially interacts with spliced/aminoacylated tRNAs. In vitro studies show that Msn5 and its homologs bind short duplex RNAs and uncharged tRNAs (Lund et al. 2004; Shibata et al. 2006). Hence, there is a contradiction between the in vitro and in vivo data. One way to rectify this contradiction would be the presence of a protein that aids the specificity of Msn5 in vivo. A candidate protein for this function is eEF1A (yeast Tef1/2).

In vertebrate cells, although eEF1A functions and localizes in the cytoplasm, it can inadvertently access the nucleoplasm during open mitosis. It has been proposed that exportin-5 binds aa-tRNA, which then recruits eEF1A to export the nuclear pools of eEF1A to the cytoplasm (Bohnsack et al. 2002; Calado et al. 2002; Mingot et al. 2013). Yeast encodes two genes for eEF1A; although this elongation factor is essential, either *TEF1* or *TEF2* can be deleted without remarkable growth defects (Grosshans et al. 2000; Murthi et al. 2010). Strains deleted for *TEF1* and *TEF2* as well as harboring a plasmid-encoding *TEF2* mutation (*tef2-1*) cause nuclear accumulation of tRNAs (Grosshans et al. 2000). However, like for *msn5*Δ and unlike for *los1*Δ cells, there is no accumulation of unspliced tRNAs in this *tef1*Δ *tef2*Δ p*tef2-1* mutant strain. Therefore, Tef1 and Tef2 appear to function in the tRNA nuclear re-export step. We also showed that the subcellular distribution of Tef1-GFP between the nucleus and cytoplasm depends on Msn5 (Murthi et al. 2010), supporting an interaction between Msn5 and Tef1/2 and demonstrating that Tef1/2 has dynamic nuclear–cytoplasmic distributions even in yeast that has a closed mitosis. Since eEF1A specifically binds aa-tRNA with nanomolar affinity and its affinity for nonacylated tRNAs is several orders of magnitude lower (Hattori and Iwasaki 1982; Faulhammer and Joshi 1987; for review, see Noble and Song 2008), we used the co-IP assays to investigate whether Tef1/2 aids the specificity of Msn5 to export charged tRNAs to the cytoplasm.

The yeast strain that we used contains an active endogenously 3Myc-tagged Tef1 (Tef1-Myc) (Supplemental Fig. S4A) and an untagged Tef2. Using anti-eEF1A (Carr-Schmid et al. 1999) and anti-Myc, we show that Msn5 copurifies with both of the comigrating Tef1-Myc and Tef2 proteins (Fig. 1E; Supplemental Fig. S4B). Thus, Msn5 interacts with Tef1/2 in vivo in a RanGTP-dependent manner. In contrast, neither Los1 nor Kap95 interacts with Tef1/2 (Fig. 1E; Supplemental Fig. S4B), providing evidence for the specificity of Tef1/2 interaction with Msn5.

### Requirement for Tef1/2 to assemble and/or stabilize Msn5–tRNA export complexes

**Figure 4.**
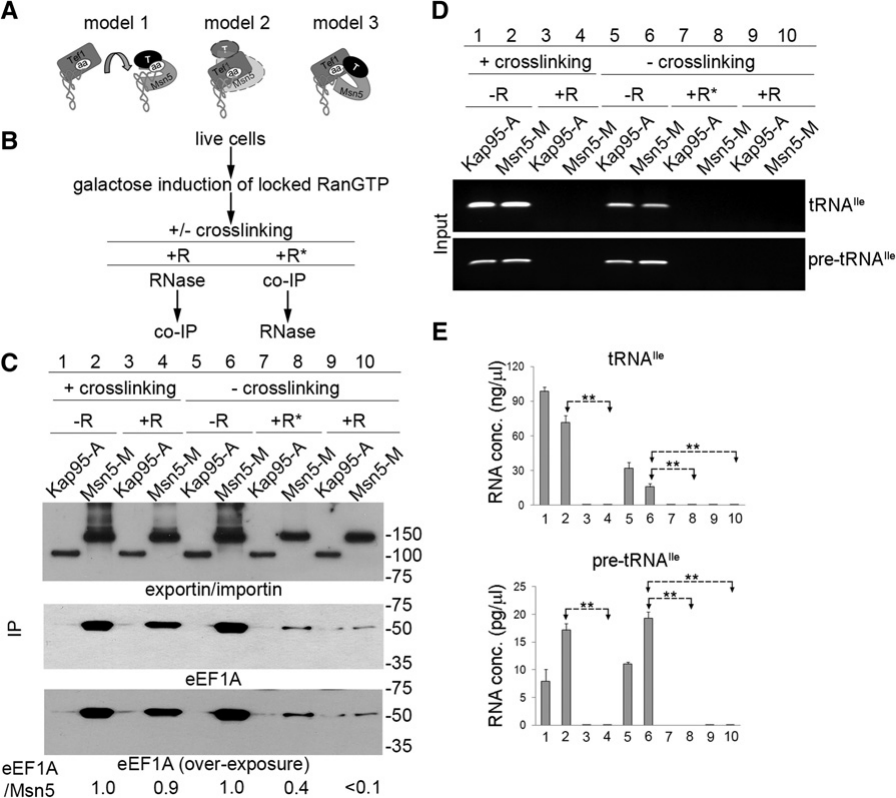
Msn5 forms a quaternary complex with Tef1/2, aa-tRNA, and RanGTP. (*A*) Hypothetical model 1: Tef1/2 hands off aa-tRNAs to Msn5. Hypothetical model 2: aa-tRNA mediates the interaction between Tef1/2 and Msn5 in complex with RanGTP to form a quaternary complex. Msn5 and Tef1/2 may compete for binding to the 3′ end of tRNA based on the predicted structure of the Msn5 vertebrate homolog exportin-5. Hypothetical model 3: Msn5 in complex with RanGTP directly interacts with Tef1/2, and Tef1/2 interacts with aa-tRNAs to form a quaternary complex. (*B*) Flow chart of experimental design. (R) The cell extract was treated with RNase A prior to co-IP; (R*) co-IP assays were performed prior to RNase A treatment. (*C*) Enriched proteins were detected by Western blot analyses using anti-protein A (*top*) or anti-eEF1A (*middle* and *bottom*) antibody. (eEF1A/Msn5) Ratio of copurified eEF1A to Msn5-M protein levels. (*D*) Input RNAs were analyzed by RT–PCR for spliced tRNA^Ile^_UAU_ and pre-tRNA^Ile^_UAU_. (*E*) Input RNAs were analyzed by RT-qPCR for spliced tRNA^Ile^_UAU_ and pre-tRNA^Ile^_UAU_. Student's *t*-test was used to calculate statistical significance of the RT-qPCR amplifications. (**) *P* < 0.0001.

Tef1 and Tef2 specifically copurify with Msn5 under all tested growth conditions (Supplemental Fig. S4C). Due to the specificity of Tef1/2 for aa-tRNAs, Tef1/2 may assist Msn5 to export charged tRNA to the cytoplasm. To provide specificity to Msn5, Tef1/2 could hand off aa-tRNA to Msn5 to form a tertiary complex, Msn5–aa-tRNA–RanGTP, for export (Fig. 4A, model 1). Alternatively, Tef1/2 might form a quaternary complex with Msn5, RanGTP, and aa-tRNA. A quaternary complex could form by Msn5 interacting with aa-tRNA, which then binds to Tef1/2 that has specificity for charged tRNAs, as previously proposed for exportin-5 (Fig. 4A, model 2; Bohnsack et al. 2002; Calado et al. 2002). Another possibility is that Msn5 directly interacts with Tef1/2, which interacts with aa-tRNA (Fig. 4A, model 3). Finally, interactions among the components could be cooperative.

To distinguish among the possibilities, we first assessed whether Msn5, Tef1/2, and tRNA exist in a single complex. We used cells containing locked RanGTP and treated with formaldehyde to cross-link Msn5 with its interacting partners. The extracts from the cells were either treated with RNase A or mock-treated prior to purification of Msn5 (Fig. 4B). Similar studies conducted with cells containing Kap95 served as negative controls. No interactions of Kap95-A and Tef1/2 were detected (Fig. 4C; Supplemental Fig. S4D, lanes 1,3). Tef1/2 copurified with Msn5-M under mock treatment (Fig. 4C; Supplemental Fig. S4D, lane 2,). Upon RNase addition, Tef1/2 maintained ∼90% of the levels relative to mock conditions (Fig. 4C; Supplemental Fig. S4D, cf. lanes 2 and 4). Under these experimental conditions, RNase treatment removed all detectable input RNAs, as assessed by Northern analysis (data not shown), RT–PCR (Fig. 4D), and RT-qPCR for tRNA^Ile^_UAU_ and pre-tRNA^Ile^_UAU_ (Fig. 4E). Thus, Tef1 and Tef2 copurified with Msn5 even when extracts were treated with RNase, supporting that Msn5 assembles into a quaternary complex consisting of Msn5, Tef1/2, RanGTP, and aa-tRNA.

Since there would be a transient interaction of Tef1/2 with Msn5 that could be stabilized by formaldehyde cross-linking, according to the hand-off model (Fig. 4A, model 1), we also conducted studies to assess formation of the Msn5 quaternary complex in the absence of formaldehyde cross-linking. Tef1/2 copurified with Msn5-M in the extracts containing RanGTP-locked mutants without cross-linking treatment (Fig. 4C; Supplemental Fig. S4D, lane 6). No Tef1/2 copurified with Kap95-A (Fig. 4C; Supplemental Fig. S4D, lane 5). Thus, cross-linking is not required for copurification of Tef1/2 with Msn5. The data argue against the hand-off model.

In an attempt to distinguish how the quaternary complex assembles, we conducted studies to deplete tRNA or Tef1/2 to assess whether interactions occur in their absence. We first conducted studies using various RNase treatments and co-IP assays. “R” indicates that the cell extract was treated with RNase A prior to co-IP, whereas “R*” indicates that co-IP assays were performed prior to RNase A treatment (Fig. 4B). When the cell extract was treated with RNase A prior to co-IP (Fig. 4B, condition R), the recruitment of Tef1/2 was reduced to <10% of the relative Tef1/2 level compared with mock conditions (Fig. 4C, cf. lanes 6 and 10), consistent with in vitro studies performed with exportin-5 (Bohnsack et al. 2002; Calado et al. 2002; Mingot et al. 2013). Surprisingly, if the Msn5-M complex was immunoprecipitated on beads first and then treated with RNase (Fig. 4B, condition R*), conditions that generate highly enriched Msn5-M compared with other cellular proteins, ∼40% of the relative Tef1/2 level was retained in complex with Msn5-M compared with mock treatment (Fig. 4C, cf. lanes 6 and 8). Assessment of RNA isolated after mock or RNase treatments showed that RNase effectively removed all detectable tRNA^Ile^_UAU_ and pre-tRNA^Ile^_UAU_ no matter the order of RNase addition and co-IP assays (Fig. 4D,E; data not shown). Although tRNA fragments associated with the complexes could have evaded detection, we interpret the data to favor the notion that Msn5 can interact with Tef1/2 in the absence of tRNA (see the Discussion).

**Figure 5.**
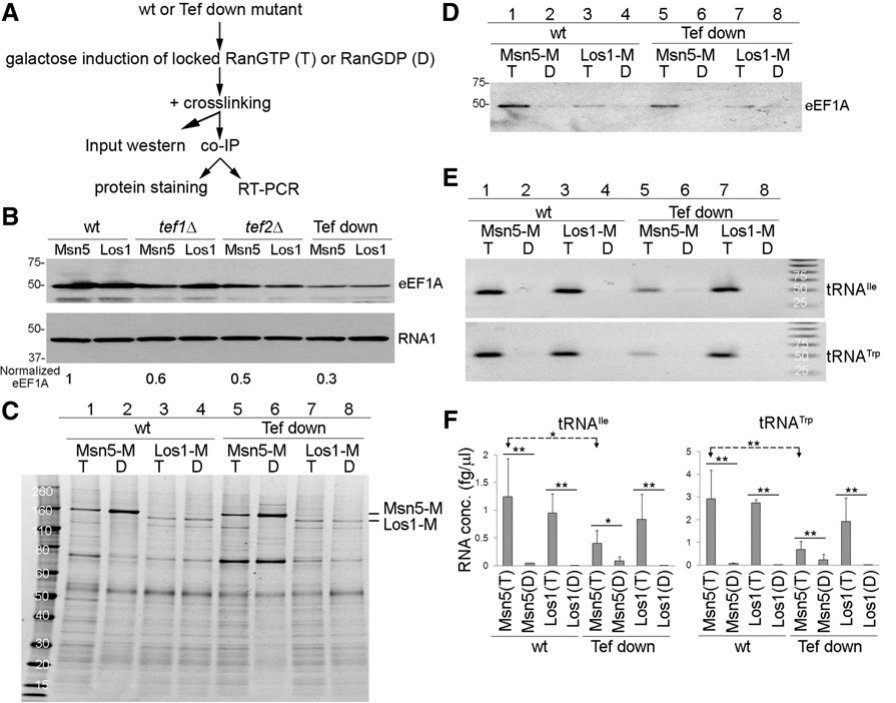
Requirement for Tef1/2 to assemble and/or stabilize Msn5–tRNA complexes. (*A*) Flow chart of experimental design. (*B*) Input proteins from wild-type (wt) cells, cells deleted for *TEF1* (*tef1Δ*) or *TEF2* (*tef2Δ*), or cells missing *TEF2* and possessing a mutant Tef1 (Tef down) were detected by Western blot analyses using anti-eEF1A. Normalized eEF1A is the ratio of eEF1A to Rna1 protein levels compared with wild-type cells. (*C*) Enriched proteins were detected by Sypro Ruby protein staining. (Lanes *1*,*2*,*5*,*6*) Msn5-M. (Lanes *3*,*4*,*7*,*8*) Los1-M. (Lanes *1*,*3*,*5*,*7*) Cells with RanGTP-locked mutants (T). (Lanes *2*,*4*,*6*,*8*) Cells with RanGDP-locked mutants (D). (*D*) Copurified proteins were detected by Western blot analyses using anti-eEF1A. (*E*) Copurified RNAs were analyzed by RT–PCR for tRNA^Ile^_UAU_ and tRNA^Trp^. (*F*) Copurified RNAs were analyzed by RT-qPCR for tRNA^Ile^_UAU_ and tRNA^Trp^. Student's *t*-test was used to calculate statistical significance of the RT-qPCR amplifications. (**) *P* < 0.005; (*) *P* < 0.05. (T) Cells with RanGTP-locked mutants; (D) cells with RanGDP-locked mutants.

If Msn5 and Tef1/2 interact and Tef1/2 acts as an adaptor for aa-tRNA, then copurification of aa-tRNA with Msn5 should depend on Tef1/2. To assess this, we altered the in vivo levels of Tef1/2. One might expect a reduction of tRNA in complex with Msn5 upon reduction of Tef1/2 levels if Tef1/2 interacts with Msn5 and brings aa-tRNA to the complex. To accomplish such experiment in vivo, we used yeast strains with mutations of *TEF1* and *TEF2*. Since Tef1 and Tef2 are essential, we constructed yeast strains that are deleted for *TEF2* (*tef2*Δ) and harbor a *tef1* mutant construct that expresses reduced protein levels, designated as the “Tef down mutant” (see the Materials and Methods). Using an anti-eEF1A antibody, Tef1 levels were reduced to 30% of the wild-type protein levels in the Tef down mutant (Fig. 5B). We then used co-IP assays using this Tef down mutant to investigate the consequences upon assembly of Msn5–tRNA export complexes (Fig. 5A).

Protein analyses showed that the levels of enriched Msn5-M proteins are similar in the wild-type and Tef down strains (Fig. 5C), whereas enriched Tef1/2 protein levels are reduced in the Tef down mutant (Fig. 5D, cf. lanes 1 and 5), likely due to reduced in vivo protein levels (Fig. 5B). In these conditions, equivalent levels of tRNA^Ile^_UAU_ and tRNA^Trp^ copurified with the control Los1-M in a RanGTP-dependent manner from both wild-type and Tef down extracts (Fig. 5E,F). In contrast, the levels of tRNAs that copurified with Msn5-M in the Tef down mutant were decreased to 25%–30% of the levels for wild-type cells (Fig. 5E,F). Thus, the assembly and/or stability of Msn5 in complex with tRNA apparently requires Tef1/2, consistent with model 3 (Fig. 4A).

## Discussion

tRNA nuclear–cytoplasmic trafficking is conserved from yeast to vertebrates. The tRNA retrograde pathway serves multiple functions, such as for complete tRNA modification (Ohira and Suzuki 2011), regulation of translation for particular amino acid biosynthetic proteins (Chu and Hopper 2013), tRNA quality control (Kramer and Hopper 2013), and delivery of retrotranscribed HIV particles to the nucleoplasm (Zaitseva et al. 2006). Thus, it is important to understand the mechanisms for tRNA subcellular dynamics. Here we developed in vivo β-importin complex co-IP assays to understand the roles of members of the β-importin family in tRNA nuclear–cytoplasmic trafficking. We provide in vivo biochemical evidence that Los1 and Msn5 have overlapping but distinct functions in tRNA nuclear export; that Msn5 assembles a quaternary complex with RanGTP, aa-tRNA, and Tef1/2; and that Tef1/2 is required for assembly and/or stability of this quaternary complex. In contrast to the predicted role of Mtr10 in tRNA nuclear retrograde import, we found no evidence for direct interaction of Mtr10 with tRNAs.

### Roles of Los1 and Msn5 in tRNA nuclear export

**Figure 6.**
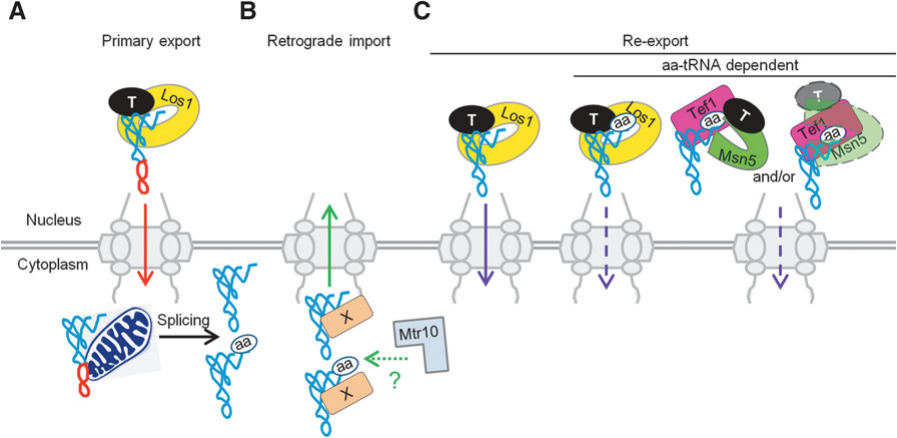
Working model of tRNA nuclear–cytoplasmic trafficking. (*A*) Los1 functions in primary tRNA nuclear export for intron-containing tRNAs. The red arrow indicates primary tRNA nuclear export. The blue drawing of tRNA indicates exon sequences; the red drawing of tRNA indicates intron sequences. (T) RanGTP. (*B*) Mtr10 may function indirectly in retrograde tRNA nuclear import. The green arrow denotes retrograde tRNA nuclear import. (X) An unknown tRNA importin; (aa) aminoacylated form of tRNA. (*C*) Both Los1 (yellow) and Msn5 (green) participate in tRNA re-export to the cytoplasm. Los1 can re-export both charged and uncharged tRNAs, but Msn5 selectively re-exports charged tRNAs. Tef1/2 aids the specificity of Msn5 for charged tRNAs by assembling or stabilizing a quaternary complex. Two organizations of the quaternary complex are presented: (*Left*) Msn5 binds Tef1/2, which binds aa-tRNAs. (*Right*) Msn5 binds aa-tRNA, which interacts with Tef1/2. The purple arrow indicates tRNA nuclear re-export. The dashed purple arrows indicate that re-export is dependent on tRNA nuclear charging.

Los1 and Msn5 serve partially overlapping but distinct roles in yeast. Los1 assembles an export complex with RanGTP and tRNA. Both intron-containing pre-tRNAs and spliced tRNAs, regardless of whether they are aminoacylated, assemble into Los1–RanGTP complexes. The tRNA nuclear export function of Los1 is likely conserved, as all studied homologs monitor the tRNA backbone and do not distinguish between intron-containing pre-tRNAs and spliced tRNAs (Arts et al. 1998b; Lipowsky et al. 1999; Cook et al. 2009). Furthermore, our results demonstrating that Los1 interacts with both aminoacylated and uncharged tRNAs are consistent with structural studies that show that *S. pombe* Xpo-t interacts with the 3′ end of tRNA but predict that Xpo-t is unlikely to distinguish between charged and uncharged tRNAs (Cook et al. 2009). Therefore, Los1 functions in the primary export of intron-containing pre-tRNAs as well as the nuclear re-export of spliced tRNAs, either charged or uncharged (Fig. 6A,C).

In contrast to the role of Los1 in both primary tRNA nuclear export and re-export, Msn5 preferentially assembles with spliced, aminoacylated tRNAs (Figs. 2, 3), documenting its role in tRNA nuclear re-export. The Msn5 vertebrate homolog exportin-5 is also reported to recruit aa-tRNAs bound to the eEF1A, thereby exporting aminoacylated tRNAs to the cytoplasm (Bohnsack et al. 2002; Calado et al. 2002; Mingot et al. 2013) even though its major role in vertebrates is to export pre-microRNA from the nucleus to the cytoplasm (Lund et al. 2004; Katahira and Yoneda 2011). Since such aminoacylated tRNAs have first been proofread by the nuclear pools of aa-tRNA synthetases, exportin-5/Msn5 provide a quality control step to assure that tRNAs capable of being aminoacylated are delivered to the cytoplasm (Lund and Dahlberg 1998).

Although Los1 and Msn5 both function in tRNA nuclear export, they cannot be the only nuclear exporters for tRNAs in yeast, as *los1*Δ *msn5*Δ double-mutant cells are viable (Takano et al. 2005). Because it is essential that tRNAs are delivered to the cytoplasm for translation, additional tRNA nuclear export pathways should exist, at least in yeast. We are currently conducting genome-wide analyses to identify the putative alternative yeast tRNA nuclear exporters.

We investigated possible mechanisms by which Msn5 specifically exports mature, charged tRNAs in vivo. Our data provide evidence that Msn5, RanGTP, aa-tRNA, and Tef1/2 assemble into a quaternary nuclear export complex. It is possible that both Msn5 and Tef1/2 contact the aa-tRNAs in the quaternary complex or that aa-tRNA binding to Tef1/2 evokes a conformational change that stabilizes Tef1/2 interaction with Msn5–RanGTP. Although we cannot distinguish between these possibilities, we favor the model in which Msn5 interacts with Tef1/2 in complex with aa-tRNA via protein (Msn5)–protein (Tef1/2) interactions (Fig. 4A, model 3) for three reasons. First, structural studies appear inconsistent with the possibility that Msn5 could first interact with aa-tRNA and then recruit Tef1/2 (Fig. 4A, model 2). Although there is no reported structure for Msn5, structural studies of its homolog, exportin-5, have led to the prediction that exportin-5 binds to the 3′ protruding end of tRNA in a similar fashion as it interacts with miRNAs (Okada et al. 2009). Since eEF1A also binds the 3′ end of tRNA when tRNA is aminoacylated (Nissen et al. 1995, 1996), in an eEF1A–aa-tRNA complex, the 3′ protruding end of aa-tRNA would be unavailable to interact with exportin-5. Second, Msn5 is known to function in nuclear export of particular phosphorylated proteins, and in vitro studies have shown that Msn5 directly interacts with its phosphorylated protein cargo, Pho4 (Kaffman et al. 1998); thus, Msn5 theoretically would possess the ability to directly bind Tef1/2. Third, our studies to assess copurification of tRNAs with Msn5 in the Tef down mutant indicate that Tef1/2 is required for robust interaction of Msn5 with tRNAs in vivo; thus, assembly and/or stability of the quaternary complex requires Tef1/2 (Fig. 5).

Future in vitro studies could be used to further distinguish whether Msn5 interacts directly with tRNA (Fig. 4A, model 2) or Tef1/2 (Fig. 4A, model 3) or, alternatively, whether the interactions are cooperative. However, appropriately post-translational modified components from yeast are required, since Msn5 is known to export phosphorylated proteins. As the ester bond between amino acids and tRNA is sensitive to alkaline hydrolysis, acid conditions throughout such in vitro studies would also be required. Finally, conformational change of Tef1/2 upon binding to aa-tRNAs would render the in vitro studies challenging.

### Questions regarding the role of Mtr10 in tRNA nuclear import

Cells bearing a mutation of *MTR10* fail to accumulate spliced tRNA in the nucleus when cells are deprived of nutrition (Shaheen and Hopper 2005; Murthi et al. 2010), presumably because retrograde tRNA nuclear import is defective. Mtr10 could function directly or indirectly in retrograde tRNA nuclear import. Surprisingly, Transportin 3 (Tnp3), the human homolog of Mtr10, binds viral capsid proteins and tRNAs in the presence of RanGTP (Zhou et al. 2011). Zhou et al. (2011) proposed that Tnp3 promotes a nuclear maturation process required for HIV-1 integration by displacing capsid proteins and tRNAs from the viral preintegration complex after nuclear entry. Despite the fact that Mtr10 is implicated in tRNA nuclear import (Murthi et al. 2010), we did not detect either RanGTP or RanGDP-dependent interactions of Mtr10 with tRNA even though the construct was functional and we could detect the known Mtr10 cargo, Npl3, in the same experiment. The most likely interpretation of the data is that Mtr10 does not directly interact with tRNAs. However, we cannot eliminate the alternatives that Mtr10 does specifically interact with tRNAs with an affinity below our detection or that the interactions of Mtr10 with tRNAs are highly unstable. If Mtr10 functions indirectly in the tRNA nuclear import step, it could do so by regulating a protein involved in tRNA nuclear import (Fig. 6B).

In sum, our working model for tRNA subcellular dynamics provides a molecular basis in which Los1 exports both mature spliced tRNAs and pre-tRNAs, whereas Msn5 has in vivo specificity for aminoacylated tRNAs. We report that assembly and/or stability of the Msn5 export quaternary complex requires Tef1/2, thereby assisting nuclear re-export of aminoacylated tRNAs to the cytoplasm (Fig. 6C).

## Materials and methods

### Yeast strains and plasmids

Most experiments used yeast strain BY4741 (*MATa his3Δ leu2Δ met15Δ ura3Δ*). Yeast strains were maintained in synthetic defined media (SC) lacking the appropriate nutritional ingredients for selection. Strain and plasmid constructions are described in the Supplemental Material.

### In vivo β-importin complex co-IP assays

Frozen ground yeast cells (0.5 g) were suspended in 5 mL of the extraction buffer (see the Supplemental Material). The soluble extract was incubated with IgG-conjugated magnetic beads for 30 min at 4°C. The magnetic beads were then collected with a magnet and washed six times with 1 mL of ice-cold extraction buffer. Protein was eluted with 0.5 M NH_4_OH and 0.5 mM EDTA by incubation for 20 min at room temperature. The eluates were lyophilized overnight using a SpeedVac (Thermo Savant). The pellets were suspended in NuPAGE LDS sample buffer (Life Technologies) and separated on a 4%–12% NuPAGE Bis-Tris gel (Life technologies) according to the manufacturer's specifications. Cell harvesting and cross-linking procedures, RNase treatment, and RNA isolation are described in the Supplemental Material.

### Western analysis

Proteins were assessed using chemiluminescence-based Western blot analysis following standard protocols as described (Chu and Hopper 2013). The concentrations of antibodies are provided in the Supplemental Material. Protein signals were quantified using ImageJ (http://rsbweb.nih.gov/ij).

### Oligonucleotides

The sequences of the oligonucleotides that we used are provided in the Supplemental Material.

### RT–PCR and RT-qPCR

RNAs were treated with Turbo DNase (Ambion) according to the manufacturer's protocol. First strand cDNA synthesis was carried out using SuperScript III reverse transcriptase (Invitrogen) according to the manufacturer's protocol. Subsequent PCR reactions were carried out using 2 µL of cDNA, and 15 µL of each PCR reaction was electrophoretically separated on 2% agarose gel. Subsequent qPCR was performed using SsoFast Eva Green Supermix (Bio-Rad) and the CFX96 instrument (Bio Rad). The PCR conditions, standard curve, controls, and data analysis are provided in Supplemental Material. The *P*-value was calculated using *t*-test calculator (http://www.graphpad.com/quickcalcs).
